# Foodborne parasites: Outbreaks and outbreak investigations. A meeting report from the European network for foodborne parasites (Euro-FBP)

**DOI:** 10.1016/j.fawpar.2018.01.001

**Published:** 2018-01-08

**Authors:** Simone M. Cacciò, Rachel M. Chalmers, Pierre Dorny, Lucy J. Robertson

**Affiliations:** a Department of Infectious Diseases, European Union Reference Laboratory for Parasites, Istituto Superiore di Sanità, Viale Regina Elena 299, Rome 00161, Italy; b Cryptosporidium Reference Unit, Public Health Wales Microbiology and Health Protection, Singleton Hospital, Swansea SA2 8QA, Swansea University Medical School, Singleton Park, Swansea, Wales SA2 8PP, United Kingdom; c Department of Biomedical Sciences, Institute of Tropical Medicine, Antwerp, Belgium; d Department of Food Safety and Infection Biology, Faculty of Veterinary Medicine, Norwegian University of Life Sciences, PO Box 8146 Dep., 0033 Oslo, Norway

**Keywords:** Foodborne parasites, Outbreaks, *Trichinella*, Cryptosporidium, Opisthorchis

## Abstract

Foodborne parasites (FBP) are a neglected topic in food safety, due to lack of awareness of their importance for public health, their biological diversity, and, for many FBP, lack of standardized detection methods, which complicates identification of the infection vehicle. The COST Action FA1408, A European Network for Foodborne Parasites (Euro-FBP), aims to limit the impact of FBP on public health by facilitating multidisciplinary cooperation and partnership among researchers, and between researchers and stakeholders. Outbreaks represent a common topic overarching specialization in one or more FBP, thus providing a platform for knowledge exchange. This report summarizes the outcomes of a meeting within the Euro-FBP consortium entitled ‘Outbreaks and Outbreak Investigations’. Recent and historical outbreaks of trichinellosis, opisthorchiasis, and cryptosporidiosis were used as examples to underline the complexity of the topic, the different foods implicated and their traceability, and the lack of standardized detection methods for some parasites. Possible solutions to overcome current limitations were also illustrated. The meeting provided an opportunity to learn from recent advances in the study of bacterial foodborne outbreaks, with an emphasis on genome analysis.

## Highlights

• Foodborne parasitic outbreaks are often difficult to identify and investigate.
• Collaboration between local health units and reference labs is essential.
• Tracing is difficult, but important for control implementation.
• Approaches in use to study bacterial foodborne outbreaks should be applied to FBP.

## 1 Background

Although foodborne parasites (FBP) are of global relevance to human and, for some parasites, animal health, compared with other foodborne pathogens they receive relatively limited attention. There are various reasons for this, including lack of awareness of the risk they pose to public health, and the comparatively long period between infection and symptoms (ranging from days to years). In addition, there is a wide disparity among FBP, not only in clinical presentations and in pathologies associated with infection, but also because of the evolutionary diversity and different diagnostic characteristics of the organisms, including protozoa of various phyla and all classes of helminths. This means that FBP research is highly fragmented, and advances in areas such as diagnostics and detection, or effects of interventions may occur separately, hampering yet further the visibility of FBP as important challenges for public health.

Specific, Europe-wide legislation on FBP is concerned with meat inspection (e.g. *Trichinella*, bovine cysticercosis), freezing requirements for fish that are to be eaten raw, and monitoring of zoonoses in food and animals, with annual reports produced by the European Food Safety Authority (EFSA) and the European Centre for Disease Prevention and Control (ECDC). However, this does not cover the full scope of relevant FBP, and national guidelines tend to be non-cohesive – not all FBP are notifiable in all countries. This again leads to the visibility of FBP being reduced.

The COST (European Cooperation in Science and Technology) Action FA1408 ‘A European Network for Foodborne Parasites: Euro-FBP (http://www.euro-fbp.org) seeks to foster interdisciplinary exchange of knowledge between diverse research groups and stakeholders working in this field. The overarching aim is to reduce the impact on human health from FBP, through establishing a risk-based control programme containing robust and appropriate protective strategies. Currently, the network includes members of over 30 European countries along with five countries not in Europe, and organizations such as EFSA and ECDC. One major public health problem common to FBP and other foodborne pathogens is outbreaks, and how they are detected, investigated, controlled and prevented.

This report highlights the main issues raised during the Euro-FBP meeting ‘Outbreaks and Outbreak Investigations’ which took place in Rome during May 2017.

## 2 Meeting report

The meeting was hosted by the Istituto Superiore di Sanità (ISS) (organizers: Simone Cacciò, Maria Angeles Gomez Morales and Marco Lalle), and immediately preceded the annual meeting of EU National Reference Laboratories for Parasitology, also hosted by ISS as the European Reference Laboratory for Parasites (http://www.iss.it/crlp/), enabling broader attendance.

### 2.1 Outbreaks and outbreak investigations

Simone Cacciò and Lucy Robertson (Chair of the Action) co-chaired the three sessions, which included: 1) presentations from four invited speakers, 2) *Cryptosporidium* outbreaks, and 3) *Trichinella* outbreaks. In addition, a poster display (not detailed in this document) gave some ad hoc insights, with particular emphasis on *Trichinella*, *Toxoplasma*, and *Cryptosporidium*.

#### 2.1.1 Session 1

Gianni Rezza (Head of the Department of Infectious Diseases, ISS) opened with *Outbreak investigations*: *the point of view of the epidemiologist*. He used the outbreak of Chikungunya virus that occurred in Italy in 2007 (Angelini et al., 2007) as an example to stress the value of collaboration between local Health Units and ISS in identifying the aetiological agent and implementing control strategies.

Dr. Rezza further emphasized that, when conditions are appropriate, vectorborne diseases, historically confined to tropical environments, can be introduced within Europe (Rezza et al., 2007). In turn, this emphasizes the need for preparedness and response to emerging infectious threats in the present era of globalization, concepts of particular relevance for FBP.

The second speaker, Pierre Dorny (Institute of Tropical Medicine, Antwerpen, Belgium) presented *Lessons from a recent outbreak of trichinellosis in Belgium*. He described an outbreak in Flanders during November 2014, in which 16 cases associated with consumption of imported wild boar were diagnosed (Messiaen et al., 2016). It was noted that the global burden of DALYs due to trichinellosis has been estimated to be 76 per billion of population per year (Devleesschauwer et al., 2015, Bouwknegt et al., 2017), with most of the burden in the WHO European region. During 2014, there were 320 confirmed human cases in this region, so those from the Belgian outbreak represent only 5%. Thus, the disease burden from trichinellosis is low, but the budget allocated for prevention is disproportionately high, with an annual estimated cost of US$ 570 million in the EU just for meat inspection. Belgium is considered a negligible risk country, and the previous outbreak, due to wild boar consumption, was in 1979 (Famerée et al., 1979). In the outbreak described, initially 10 cases were admitted to hospital in late November 2014, all with fever, peri-orbital swelling, muscle pain and eosinophilia. *Trichinella spiralis* infection was confirmed, and six more patients were identified. All 16 cases had eaten wild boar meat in three restaurants in Flanders in early November. Two exposure groups were identified – severe exposure cases (n = 10) had eaten a full portion of slowly roasted wild boar tenderloin, while those with mild exposure (n = 6) had eaten either small amounts of the tenderloin or wild boar stew. Interestingly, three people who, according to their food intake, had been exposed at the same time neither developed symptoms nor became seropositive.

Patients with mild exposure reported a significantly longer incubation period (19–24 days) than those with severe exposure (6–21 days). The mean period of hospitalization (n = 10) was 14 days (range 2–75 days), and three cases required treatment in an intensive care unit. In one patient, signs and symptoms persisted for several months.

A single distributor of wild boar meat from a supplier in North-Eastern Spain was implicated as the likely source of infected meat, but examination of meat from this and other batches was negative for *Trichinella*. This points to a common problem with investigation of outbreaks of foodborne parasitoses, namely, that by the time the diagnosis is made, the actual food component implicated has been either eaten or discarded. In such cases, swift action by local agencies is critical, particularly with regard to informing others who may also have been exposed to the infection source.

In conclusion, although trichinellosis is a rare disease in Western Europe, it remains a threat to foodchain safety despite control measures taken at the European level. Timely identification of an outbreak and tracing of implicated food, are hampered by complex supply chains. A transnational early warning system will be important to alert the appropriate authorities, who can take swift action and control further spread of an outbreak.

Edoardo Pozio (Head of the European Union Reference Laboratory for Parasites, ISS) presented on *Outbreaks of opisthorchiasis in the European Union* that occurred in Italy between 2007 and 2011 (Armignacco et al., 2008; Traverso et al., 2012; Pozio et al., 2013). He described the complex lifecycle of these trematodes, their geographic distribution, and the demonstrated association with human cholangiocarcinoma. In Europe, the main circulating species is *Opisthorchis felineus*, and humans acquire the infection by consumption of raw or undercooked freshwater fish of the family Cyprinidae. The main acute symptoms are fever, abdominal pain, and diffuse myalgia, and patients have marked leukocytosis, eosinophilia and elevated levels of alanine aminotransferase.

In the 2007 outbreak, the epidemiologic investigation of two male index cases revealed that 25 days prior to presentation they, and 34 men from different villages, attended a dinner in a private home, and consumed marinated tench and whitefish fillets from Bolsena lake. Only those who had consumed tench fillets acquired the infection (22 people, 59% attack rate). Dr. Pozio emphasized that the fish was frozen at −10 °C for 3 days, and then marinated before consumption.

In the 2009 outbreak, the index case was a woman who consumed fresh, raw tench fillets during a gastronomic event in the town of Bomarzo (Latium region) on December 8. As the event was by invitation only, all 44 participants were traced after 4–5 months (April–May 2010), and 12 had experienced symptoms of unknown etiology in late December 2009 and early January 2010. The investigation revealed that the catering company that organized the event had purchased tench from Bolsena Lake.

In the 2010 outbreak, the index case was admitted to hospital in Valle d'Aosta region on August 15, and then 10 additional people were hospitalized from August 22 to 26. Further cases (n = 49) were diagnosed between September and December. The epidemiologic investigation revealed that all 60 cases had attended a gastronomic event for 93 people in the Challand-Saint-Anselme village (Aosta region) on July 24 (attack rate, 64.5%). One of the infected persons was a tourist from the Netherlands. The organizers of the event served marinated tench fillets (whitefish in the brochure) purchased in Bolzano, but originally marketed by a fish company operating in the Bracciano Lake (Latium region). Importantly, according to the Italian regulation, boxes containing tench must be labelled with a warning stating that the fish must be frozen before consumption. These labels were lost during transportation.

The 2011 outbreak was the largest, involving 80 people. The index case was hospitalized on August 24, and 18 additional people were admitted to the same hospital from August 25 to 31. Between September and October, 61 additional cases were diagnosed. Overall, 56 patients were symptomatic, and 24 were asymptomatic. The epidemiologic investigation revealed that all infected persons had a meal at a restaurant on the Bolsena lakeshore, on July 22, 2011. During that day about 500 people had eaten raw marinated fish, but only 100 could be traced. Among the infected persons, two were from Austria and seven from the Netherlands. As in the 2010 outbreak, fresh, raw tench fillets were served at the restaurant because of lower cost and greater availability during the summer, when the demand for whitefish exceeds supply. Dr. Pozio also emphasized that many tourists may be unable to distinguish the taste of whitefish from tench, allowing for the possibility of food fraud.

Dr. Pozio concluded by stressing that: 1) the clinical signs and symptoms of opisthorchiasis are not pathognomonic, 2) most physicians in EU are not aware of this disease, and 3) about one third of people infected with *O*. *felineus* are asymptomatic. Therefore, an unknown number of persons in the EU may be living with cryptic and chronic infections, and may be at risk of developing cholangiocarcinoma.

The session ended with a presentation from Dr. Morabito (Head of the European Union Reference Laboratory for VTEC, ISS) on *Applications of NGS to outbreak investigations*: *VTEC and beyond*. The focus was on Shiga-toxin producing *Escherichia coli*, a cause of haemorrhagic colitis and haemolytic uremic syndrome (HUS) in humans. Dr. Morabito described the major outbreak that occurred in Germany in 2011, and recalled that this was the first investigation where the genome of an isolate was fully sequenced using NGS technology (Mellmann et al., 2011). Genome analysis showed that the outbreak strain was the result of a combination of Enteroaggregative and Shiga toxin producing *E*. *coli*, and had virulence factors from both types of bacteria, explaining the particularly high virulence.

Dr. Morabito also presented an investigation of a cluster of HUS cases in Italy to underline the value of NGS data in the management of this event. He showed how a comparative genomic approach enabled confirmation of the epidemiologic link between some cases and the potential reservoir (cattle), while excluding this link for other cases.

Applications of NGS in the field of parasitology are still limited, but there are good examples, at least for protozoa, to show how genome analysis can provide important insights by comparing outbreak versus non-outbreak strains, which may eventually lead to the development of more informative genotyping tools (Beser et al., 2017; Sikora et al., 2017).

#### 2.1.2 Session 2 (*Cryptosporidium*)

Rachel Chalmers (Head of the Cryptosporidium Reference Unit, Swansea, UK) posed the question “*How do you know if there*'*s an outbreak*?” Her starting point was the different laboratory practices across the EU that partly contribute towards quite large differences in infection ascertainment, as described for *Cryptosporidium* and other pathogens by Haagsma et al. (2013). As well as affecting our ability to assess the burden of illness or measure temporal or seasonal variations in the number of cases, these differences may also hamper our abilities to identify, monitor and investigate outbreaks, especially when they occur across international borders. Dr. Chalmers then presented two UK-based studies that investigated how outbreak detection can be improved. The first was based on a questionnaire survey of health protection teams (HPTs) and showed that outbreaks were more likely to be reported by HPTs (i) where exceedance monitoring was in place, (ii) that always administered a risk factor questionnaire, and that had a structured system in place for detection of common exposures (Chalmers et al., 2016). The second investigated whether the use of SaTScan™, a free software that analyses spatio-temporal data, could improve outbreak detection; SaTScan modeling identified an additional 3 outbreaks (among a total of 8) in a 2-year period in a population of 2.23 million (Briggs et al., 2014). Finally, Dr. Chalmers explored the use of *Cryptosporidium* genotyping to assist in identification of outbreaks, and showed how emergence of a particular *C*. *parvum* genotype in one region, followed by a national peak in that same genotype, was used for hypothesis generation. Prospective molecular surveillance for epidemic response would be reliant on continuous genotyping of all or sentinel cases.

In conclusion, Dr. Chalmers suggested that *Cryptosporidium* outbreak recognition could be enhanced across Europe by improved diagnosis, notification, and investigation of cases, the use of mathematical modeling approaches, greater use of genotyping, standard response from HPTs and improved food chain analysis. She suggested that the FoodChain-Lab open-source software (Weisser et al., 2016), which has been used for investigating food-related outbreaks in Germany since 2011 (including the 2011 outbreak mentioned by Dr. Morabito), could be applied to parasites.

Next, Heidi Enemark (National Veterinary Institute, Norway) described three outbreaks of cryptosporidiosis in Denmark. The first was a nosocomial outbreak in AIDS patients in 1989, and ultimately may have contributed towards the deaths of 8 patients; the vehicle was ice from an ice machine, contaminated by a diarrhoeic, psychotic patient with cryptosporidiosis picking out ice for cold drinks (Ravn et al., 1991). The second outbreak involved 99 employees eating at a company canteen, with apparent contamination of the salad bar (Ethelberg et al., 2005). Finally, Dr. Enemark presented an outbreak among veterinary students associated with obstetrics and large animal practice; cryptosporidiosis outbreaks are common among such students, but this one would probably not have been detected had the students not demanded an investigation. It was concluded that with a lack of routine diagnosis, and an absence of awareness/surveillance/notification, outbreaks of cryptosporidiosis in Denmark (and elsewhere) are likely to continue, and remain undetected.

#### 2.1.3 Session 3 (*Trichinella*)

The final session focused on three outbreaks of trichinellosis, all with a Serbian connection. Helena Yera (Reference Laboratory for Human Trichinellosis, Paris, France) described a recent French outbreak in February–March 2017. This involved families in Serbia (two cases) and France (8 cases), with pork imported from Serbia consumed in France. Analysis revealed 51 *Trichinella spiralis* larvae per gram in sausages and 62 larvae per gram in dried meat. Specific problems associated with detection of this international outbreak included medical/clinical difficulties, including delays in consulting a physician and initial misdiagnosis, and a language barrier.

Jelena Petrovic (Scientific Veterinary Institute “Novi Sad”, Novi Sad, Serbia) described an outbreak investigation in 2013 in which four people, all males aged between 19 and 25 years, were suspected to have trichinellosis based on symptoms and serology, and 3 were hospitalized. They had bought and barbecued sausages for a May holiday picnic; although none of the specific food was available for analysis, a raid by veterinary inspectors on the butcher shop where the sausages had been purchased resulted in detection of *T*. *spiralis* larvae in two samples of traditional smoked meat products. The butcher confessed that he illegally produced raw sausages and traditional smoked sausages at home. As a result, the shop was closed down and the butcher prosecuted. Dr. Petrovic concluded that the prevalence of *Trichinella* in pigs in Serbia is high, and the risk for human infection is affected by poor socioeconomic conditions, insufficient education of farmers and food business operators, lack of veterinary controls and improper disposal of dead animals, exacerbated by illegal actions of irresponsible producers.

The final presentation by Sasa Vasilev (National Reference Laboratory for Trichinellosis, University of Belgrade, Serbia) focused on a large outbreak of *Trichinella britovi* associated with consumption of wild boar meat. Of the 273 people exposed, 114 were diagnosed with trichinellosis, and 19 were hospitalized. The infected meat was consumed between late December 2016 and mid-January 2017. It appeared that hunted wild boar was not always subject to veterinary controls.

## 3 Discussion

Outbreaks caused by FBP continue to be difficult to identify and even more challenging to investigate. The weakest link in current practice for delayed or missed recognition of foodborne outbreaks will vary by parasite, but may include low index of suspicion, limited knowledge from healthcare practitioners, inability to obtain the correct samples, and lack of use of the most appropriate tools for analysis. The pyramidal “iceberg” of lack of case detection has been described in various articles, and not only for Europe (Allos et al., 2004).

Investigation of outbreaks caused by other pathogens, and by FBP not discussed here, is increasingly relying upon new approaches, particularly genome sequencing. Currently, national strategies for implementation of genome sequencing tend to focus on bacterial and viral pathogens, but the inclusion of FBP should be encouraged, as approaches for parasites are being developed. Finally, in the global food chain, it is important to be aware that no country is an island.
